# Changes in physical activity across retirement: a compositional data analysis approach in a Swedish cohort study

**DOI:** 10.1186/s11556-025-00395-6

**Published:** 2025-12-19

**Authors:** Lawrence B. Sacco, Robin S. Högnäs, Javier Palarea-Albaladejo, Pasan Hettiarachchi, Magnus Svartengren, Hugo Westerlund

**Affiliations:** 1 Division of Psychobiology and Epidemiology, Department of Psychology, Stockholm University, Stockholm, Sweden; 2 Department of Computer Sciences, Applied Mathematics and Statistics, University of Girona, Girona, Spain; 3 Department of Occupational and Environmental Medicine, Uppsala University Hospital, Uppsala, Sweden; 4 Department of Medical Sciences, Uppsala University, Uppsala, Sweden

**Keywords:** Retirement, Older adults, Compositional data analysis, Physical activity, Sedentary behavior, Sleep, Device-based measurements

## Abstract

**Background:**

Retirement is a major life transition that can alter patterns of movement behaviors (physical activity, sedentary behavior and sleep). While some studies indicate an increase in physical activity post-retirement, others report a rise in sedentary behavior. However, evidence is lacking on how individuals re-allocate time among movement behaviors, particularly using analytical approaches that account for the co-dependence of 24-hour time-use data. Furthermore, little is known about how pre-retirement occupational physical activity (OPA) levels influence physical activity after retirement. This study examined changes in the relative time spent in sleep, sedentary behavior (SB), light physical activity (LPA), and moderate-vigorous physical activity (MVPA) over retirement, and how these changes vary by pre-retirement OPA levels.

**Methods:**

Data were drawn from the Swedish Retirement Study, which followed 112 participants (47 men, 65 women; age: 60–72) at three timepoints during the retirement transition. Movement behavior and sleep data were collected over a week-long period using thigh-worn accelerometers and wrist-worn actigraphs. Compositional data analysis (CoDA) was employed to account for the co-dependent nature of 24-hour time-use data. Multivariable linear mixed models, adjusted for sociodemographic and health covariates, were used to evaluate the associations between retirement, OPA tertiles, and movement behaviors.

**Results:**

In the overall sample, changes in movement behaviors mainly involved sleep. However, substantial variation was observed across OPA tertile groups. The sleep-to-wake time ratio increased in the high OPA group and, to a lesser extent, in the medium OPA group. Regarding physically active and sedentary time, a convergence between the high and low OPA groups was observed, as pre-retirement differences diminished. Specifically, the ratio of physically active time to SB decreased in the high OPA group and increased in the low OPA group.

**Conclusions:**

The findings indicate that pre-retirement OPA is a significant factor in understanding changes in movement behaviors during the retirement transition. The reduction in post-retirement physical activity among high-OPA workers may represent a healthier rebalancing rather than a decline, which aligns with the “physical activity paradox” and the “Sweet-Spot Hypothesis”. This evidence highlights the need for tailored interventions for retirees, particularly those from physically demanding occupations.

**Supplementary Information:**

The online version contains supplementary material available at 10.1186/s11556-025-00395-6.

## Background

Retirement is a significant life transition that potentially reshapes older adults’ daily activity and time-use patterns [1]. Most notably, retirees either exit the paid labor force or significantly reduce their participation in it. The social organization of life can diversify, with the lives of many older adults not primarily revolving around work but rather being mixed with other activities and roles that can impact available time and level of physical activity. Furthermore, adults in their 60s, who are at typical retirement ages, often experience major life changes related to their health. Chronic disease incidence, for instance, rises after age 50, with comorbidities becoming more prevalent from age 65 onward [2, 3].

For better or worse, retirement can have significant implications for health as work time is by necessity re-allocated to potentially sedentary, physically active, or sleep time, making this life transition a period of risk and opportunity in terms of setting new health habits [4]. In the context of population aging, physical activity among aging adults plays an important role in the overall health of a population. Research shows that maintaining a physically active lifestyle and minimizing sedentary behavior reduces the risk of a range of chronic diseases and is associated with better mental health, quality of life, and cognitive functioning [5–8]. Therefore, in order to promote healthy aging in later life, it is important to understand how retirement might impact levels of physical activity and sedentary behavior.

The transition to retirement may allow those in mostly sedentary jobs to move more and decrease sedentary behavior, as individuals may view retirement as an opportunity to become more physically active [9]. The Organisation for Economic Co-operation and Development (OECD) reports that more than 1 in 3 adults in Europe do not meet the physical activity guidelines set by World Health Organization (WHO), with decreased occupational activity being one of the drivers of inactivity [10]. Conversely, those in physically demanding jobs may regard retirement differently, for example, as an opportunity to rest more and recover. It is, however, unclear how changes in physical activity and sedentary time play out over time for retirees in different occupations.

Occupational Physical Activity (OPA) is physical activity that is built into the job. It can include tasks that require walking, lifting, carrying, standing, and manual labor. By contrast to leisure-time physical activity, which generally includes aerobic activities and recovery for rest and training effects, OPA can be static and straining on the cardiovascular system [11, 12]. As described by the “physical activity paradox”, OPA and leisure-time physical activity can have contrasting effects, with the former potentially leading to detrimental effects on health [13–15]. Evidence indicates, for example, that high levels of OPA may increase the risk of musculoskeletal disorders, cardiovascular disease, and higher mortality [11, 14, 16], although this may be dependent on the type and intensity of the physical work, and the person’s age and fitness level [17–19].

This heterogeneity is acknowledged by the “Sweet-Spot Hypothesis,” which emphasizes achieving a “healthy balance” of movement behaviors within the 24-hour day, including sufficient sleep, sedentary, and physically active time [20]. In practical terms, an increase of, for example, light physical activity may have different health consequences if it is compensated by a decrease of sleep, more vigorous physical activity or sedentary time [21, 22]. Consequently, the traditional “sit less–move more” recommendation might be suited only for workers in sedentary occupations, as it would bring them towards their 24-hour movement behavior “sweet-spot”, but not necessarily for those in physically demanding occupations [20]. These considerations are particularly relevant for retirees as they would imply varying changes in movement behaviors over the retirement transition depending on prior OPA levels.

While previous research has shown that retirement is generally associated with changes in physical activity and sedentary behaviors [23–25], inconsistent findings have been reported with persisting research gaps. Firstly, previous studies have yielded mixed findings when using self-reported measures of sedentary behavior, while device-based measures may be more reliable [26]. For example, studies have suggested both decreases in sitting time and increases in sedentary activities (e.g., watching TV) over retirement [25]. Secondly, few studies have considered the relative nature of individuals’ movement behaviors through a compositional approach to model movement behaviors, including sleep, light physical activity (LPA), moderate to vigorous physical activity (MVPA), and sedentary behavior (SB), over a 24-hour period [1, 27]. Findings based on compositional approaches are needed to better understand movement behaviors and inform physical activity guidelines [22]. Finally, the association between retirement and movement behaviors is likely multifaceted, varying according to individual circumstances, such as socioeconomic status, environmental factors, and one’s physical activity levels while in paid work [28]. Previous research has suggested that individuals retiring from sedentary or standing occupations increase their self-reported physical activity post-retirement, while those in more physically demanding occupations reduce it [4]. To date, there is a lack of research assessing the variation in movement behaviors across retirement by pre-retirement levels of OPA using device-based measures and compositional approaches.

The compositional data analysis (CoDA) methodology, as applied in the current paper, provides a consistent and holistic framework for assessing changes in physical movements across the retirement transition [29]. CoDA recognizes that time-use data are inherently multivariate and relative, since they refer to fractions of time allocated across different activities out of a total time available (typically 24 hours). Hence, the measurements of the use of time are intrinsically co-dependent and convey relative information. By examining time-use patterns as a whole, rather than focusing on individual activities in isolation, CoDA allows for a more comprehensive understanding of the changes in physical activity and SB that occur from pre-retirement to post-retirement [22].

This study contributes to the literature by assessing how the "Sweet-Spot Hypothesis" relates to OPA vis-à-vis the transition into retirement. To this end, we examine the following questions: (1) How do physical behavior compositions (i.e., sleep time, SB, LPA and MVPA) change across the retirement transition? (2) How does pre-retirement OPA influence physical behavior across the transition to retirement? These insights can help to inform public health guidelines for promoting healthy aging, and help individuals make informed decisions about how to maintain their health and wellbeing as they move into retirement.

## Data and methods

### Data

Wrist actigraphy, thigh accelerometer, diary, and survey data from the Swedish Retirement Study (SRS) were used. The SRS consists of data on health and movement behaviors from 119 participants (ages 60 to 72) who have been followed across the retirement transition [30]. Participants were recruited from two larger survey studies: the 2016 and 2018 waves of the Swedish Longitudinal Occupational Survey of Health (SLOSH) and the Aging at Work study [30, 31]. Invitations to participate in the SRS were sent to 1078 people over age 59, still in paid work, and who were planning to retire within two years. To be included in the cohort as retirees, participants had to reduce working hours by at least 50% of full-time and work 25% or less of full-time. Those who worked nightshifts (10:00pm–6:00am), were on sick leave, or unemployed for more than 25% of the time within the six months before retirement or more than three months over the last year were excluded. Further details about recruitment of the SRS are available in Garefelt et al. 2021 [30].

Data were collected at three time points: approximately six months pre-retirement, and at six (± 2) and 18 (± 2) months post-retirement. At each time point, data collection took place over one week and involved a general questionnaire, a diary questionnaire, a wrist actigraph (wActiSleep-BT or wGT3X-BT, ActiGraph Ltd), and thigh and chest triaxial accelerometers (Axivity AX3, Axivity Ltd). Participants were mailed the electronic devices, questionnaire, and diary, along with detailed instructions for completing the questionnaires and placing the devices (additional over-the-phone guidance was available). The instructions recommended wearing the accelerometers for approximately four days (including two weekdays and two weekend days) and the wrist actigraph continuously throughout the week. Participants were asked to complete the general questionnaire, which collected information on occupation, health and health behaviors, at the beginning of the measurement week. The diary questionnaire, which was to be completed every morning and evening during the measurement week, collected information on device wear periods (dates and times), sleep schedules, the start and end times of up to three work spells per day, as well as data on stress, sleep quality and alertness.

Concerning the analytic sample, all days with valid data for movement behaviors over the 24-hour period were initially selected, resulting in 936 days from 119 individuals. Data from 14 days were excluded due to inconsistencies in the collection of actigraph and accelerometer data. Four participants with missing information on work periods were excluded. Data from 41 days that followed a seasonal clock change were excluded as these affected the validity of the overall measurements. In total, 860 days of data, distributed over 301 collection weeks (pre and post-retirement follow-ups) from 112 participants, were included in the analytic sample.

### Sleep and movement behaviors

Wrist actigraphy on the non-dominant arm was used to derive sleep time, defined by the time interval between onset of sleep and final awakening. Actigraphy recordings were processed with the software from the device manufacturer (Actilife) using the Cole-Kripke algorithm with visual inspection for quality control, as previously noted [30]. For one individual, who was missing simultaneous accelerometer and actigraphy recordings, sleep periods reported in the daily diary were used instead, as individuals reported the time they went to sleep and turned off the light, and the time when they woke up every day.

Measures of time spent in a state of SB, LPA, or MVPA during the day were derived from recordings from the thigh-worn accelerometer. Triaxial raw data from the accelerometers were processed using an automated version of the Acti4 software, ActiPASS [32–35]. ActiPASS applied both a device specific and participant specific calibrations, allowing automatic correction of any orientation and placement issue of the accelerometers [32, 34, 36]. While non-wear periods were automatically identified by the data processing procedure in ActiPASS, manual inspection by one of the authors ensured the correct determination of the start and end of recording periods, as well as the larger non-wear period gaps that occurred due to participants being instructed to wear the accelerometer for two weekdays and two weekend days.

Reference positions, corresponding to fixed postural positions and moving states, were identified. Following the guidelines set by the Sedentary Behavior Research Network [37], these were grouped into each of the movement behaviors: lying and sitting postures were made to correspond to SB; standing, moving, and slow walking with a cadence of < 100 steps per minute corresponded to LPA; and fast walking with a cadence of 100 steps per minute or more, stair walking, running, cycling, and other behaviors with movements corresponded to MVPA. The ActiPASS algorithm assigned portions of the days into postures, with sleep time periods identified by the above-mentioned wrist actigraphy. Thus, each 24-hour day, starting at midnight, could be subdivided into time spent on either sleep, SB, LPA or MVPA.

### Covariates

The pre-retirement OPA variable was derived from accelerometer and diary data. Self-reported diary entries identified paid work spells during waking time. Participants indicated whether they worked each day and, if so, reported the clock times when they started and finished paid work, including the times for separate work spells within a single day. This division was applied during the accelerometer data processing step to quantify the time spent in SB, LPA, and MVPA during and outside of work periods. Consistent with previous studies [12, 15], OPA tertiles were calculated for each individual based on the percentage of work time spent in physical activity (LPA and MVPA). The mean percentages of OPA were 18.7% for the low, 36% for the medium, and 63% for the high tertile group.

Sociodemographic and health variables were included in the multivariable models as potential confounders. Information on age (continuous) and gender (man/woman) was drawn from the LISA (the longitudinal integrated database for health insurance and labour market studies) administrative registers. Marital status at baseline was derived from the general questionnaire, as participants were asked whether their marital status corresponded to being either married/cohabiting, in a non-cohabiting relationship, or single. This measure was dichotomized (0 = married/cohabiting; 1 = neither married nor cohabiting).

Occupational social class was derived from the general questionnaire. Participants reported their main occupation which was classified according to the Swedish Standard Classification of Occupations (SSYK-12), based on the International Standard Classification of Occupations (ISCO-08). While all categories of this variable are displayed in the descriptive findings (Table 1), this measure was dichotomized when included in the multivariable models (0 = Nonmanual, ISCO 1–4; 1 = Manual, ISCO 5–9) as done in a previous study [38].

**Table 1:** Pre-retirement baseline characteristics of the study sample (*N* = 112)

Variables	*n* (%) or Mean (SD)
Gender	
Men	47 (42.0%)
Women	65 (58.0%)
Age	64.86 (2.00)
OPA, mean of percentages (SD)	39.4 (20.71)
Marital status	
Married/cohabiting partnership	91 (81.2%)
Single or non-cohabiting	21 (18.8%)
Occupational social class	
1-Managers	11 (10.2%)
2-Occupations requiring advanced level of higher education	44 (40.7%)
3-Occupations requiring higher education	22 (20.4%)
4-Administration and customer service clerks	14 (13.0%)
5-Service, care and shop sales workers	7 (6.5%)
6-Agricultural, forestry and fishery workers	0 (0.0%)
7-Building and manufacturing workers	8 (7.4%)
8-Mechanical manufacturing and transport workers, etc.	1 (0.9%)
9-Elementary occupations	1 (0.9%)
Missing	4 (3.6%)
Chronic disease(s) affecting life	
none	44 (39.3%)
1 chronic disease	34 (30.4%)
>1 chronic diseases	34 (30.4%)

Mean (SD) for continuous variables and n (%) for categorical ones. *SD* standard deviation

A chronic disease measure was derived from participants answering the question “Has a doctor ever told you that you have …” for 23 chronic ailments (e.g., angina, cerebrovascular disease, diabetes, fibromyalgia, hypertension, arthritis, osteoporosis, and Parkinson’s disease). Response options were: “No”, “Yes, but doesn’t affect life”, “Yes, affects my life a little” and “Yes, affects my life a lot”. As previously done [38], the constructed variable consisted of three categories: “None”, “one disease affecting life” and “multiple diseases affecting life”.

### Statistical analysis

We used CoDA to model and analyze the movement behavior data comprising four parts: sleep, SB, LPA, and MVPA. CoDA methods naturally deal with the codependency between data parts and the total time constraint [29, 39, 40]. In brief, the focus is set on the relative information conveyed by the data by representing the original composition using unconstrained log-ratios. In particular, isometric log-ratio (ilr) coordinates have been commonly used in physical activity and time-use epidemiological research. These were obtained in the current study through the sequential binary partition procedure [40]. This allowed us to define log-ratios representing contrasts of interest between parts, or groups of parts, of the movement behavior composition. Thus, three ilr-coordinates resulted from the 4-part composition, expressed as

$$\begin{aligned} &\:{ilr}_{1}=\sqrt{\frac{3}{4}}ln\frac{Sleep}{\sqrt[3]{SB\times\:LPA\times\:MVPA}},\:\:\:\:\\&{ilr}_{2}=\sqrt{\frac{2}{3}}ln\frac{SB}{\sqrt{LPA\times\:MVPA}}\:\:\mathrm{a}\mathrm{n}\mathrm{d}\:\:\\&{ilr}_{3}=\frac{1}{\sqrt{2}}ln\frac{LPA}{\sqrt{MVPA}}. \end{aligned}$$

These variables, which are defined on the ordinary unconstrained real space, capture how much larger or smaller movement behaviors are in the numerator compared to those in the denominator (averaged by their geometric mean). Thus, $\:{ilr}_{1}$ represents the contrast between time spent sleeping to wake time (i.e. the average of SB, LPA and MVPA), $\:{ilr}_{2}$ confronts time in SB to all physical activity, and $\:{ilr}_{3}$ is comparing LPA against MVPA time.

The descriptive analyses of movement behavior composition include the calculation of the compositional geometric mean, as a central tendency measure, and the variation matrix to summarize the structure of relative variation among behaviors. The former consists of computing the geometric mean of each part and closing the resulting vector to add up to the predetermined total time. The latter comprises the calculation of the variances of all pairwise log-ratios, which measures their proportional degree of co-dependence in terms of proportionality. Thus, values near zero indicate that a log-ratio remains relatively constant and, hence, the behaviors involved are nearly proportional. Additionally, the total variance (i.e., the sum of all pairwise log-ratio variances) was decomposed into contributions from each behavior. Differences between time-use profiles across the retirement transition were assessed using geometric mean barplots.

Random intercept linear mixed models [41] were fitted by restricted maximum likelihood to assess how measurement occasion influenced movement behaviors. These models included individual ID as a random effect to account for the within-subject correlation across repeated measurements over time and heterogeneity between individuals due to different baseline levels. Two sets of analyses were performed for each research question. For each set of analyses, separate models were fitted to each ilr-coordinate $\:{ilr}_{i}$, with $\:i=1,\:2,\:3$, serving as the dependent variable. In the first set of analyses, retirement status was treated as a fixed characteristic in the mixed models, followed by post hoc contrasts of differences across retirement groups. In the second set of analyses, the interaction between OPA and retirement status was added to examine any effect modifying role of OPA.

Data management was largely done using Stata 16, whereas CoDA and modelling were performed using custom computer routines on the R system for statistical computing v4 (R Foundation for Statistical Computing, Vienna, Austria). Where applicable, *p*-values derived from multiple statistical testing were adjusted for false discovery rate (FDR) [42]. Statistical significance was generally concluded at the 5% significance level. Multiple imputation by chained equations (MICE) was used to impute the missing values in the occupational social class variable, using Rubin’s rule when estimating model parameters from the imputed datasets [43]. The MICE method used predictive mean matching to produce 20 imputed datasets through 50 iterations with the following variables: the three ilr-coordinates, gender, age, marital status, chronic diseases affecting life, and percentage of work time spent in physical activity.

## Results

### Descriptive analysis

Table 1 shows the characteristics of the study sample at the pre-retirement baseline. The average age of the participants was 64.9 years, with 58% women. Most participants (81%) were married or in cohabiting partnerships, and the majority were in either managerial (10%) or occupations requiring higher education (61%). A considerable proportion of the sample reported one or more chronic diseases that were affecting their life (61%).

Compositional descriptive statistics across retirement are shown in Table 2. On average, participants spent the most time in SB at all timepoints: 10.7 h/day (pre-retirement), 10.35 h/day (6 months post-retirement), and 10.55 h/day (18 months post-retirement). Following SB, participants spent the most time on sleep (range: 7.52–8 h/day), then LPA (4.41–4.6 h/day) and finally MVPA (1.15–1.2 h/day). In the pre-retirement period, the highest co-dependence was between LPA and SB (log-ratio variance: 0.31), with LPA also contributing the most to the total variance (33.5%). For both post-retirement timepoints, the largest co-dependance was between MVPA and SB (0.44 and 0.32), with MVPA contributing the most to the total variance (42.7% and 40.2%). This suggests that while before retirement LPA plays a larger role in determining interindividual compositional variance between individuals, MVPA does so after retirement.

**Table 2:** Compositional means and variation of movement behaviors

		Variation Matrix				
	Mean min/day (%)	Sleep	SB	LPA	MVPA	Contr. tot. var. (%)
Pre-retirement (*N* = 112)						
Sleep	451 (31.3)	0	0.07	0.18	0.17	11.9
SB	642 (44.6)	0.07	0	0.31	0.23	28.5
LPA	276 (19.1)	0.18	0.31	0	0.18	33.5
MVPA	72 (5.0)	0.17	0.23	0.18	0	26.0
6 months post-retirement (*N* = 104)						
Sleep	480 (33.4)	0	0.06	0.16	0.33	10.6
SB	621 (43.1)	0.06	0	0.28	0.44	25.7
LPA	269 (18.7)	0.16	0.28	0	0.27	21.0
MVPA	70 (4.8)	0.33	0.44	0.27	0	42.7
18 months post-retirement (*N* = 90)						
Sleep	473 (32.9)	0	0.05	0.14	0.23	10.0
SB	633 (44.0)	0.05	0	0.23	0.32	24.9
LPA	265 (18.4)	0.14	0.23	0	0.23	24.9
MVPA	69 (4.8)	0.23	0.32	0.23	0	40.2

Compositional geometric means of movement behaviors are shown adjusted to sum to 1440 min and in the respective percentage. Percentage of contribution to total variance are calculated from clr (centered log-ratio) transformed data. *Abbreviations*: *Contr*. *tot*. *var*. contribution to total variance

**Fig.1:**
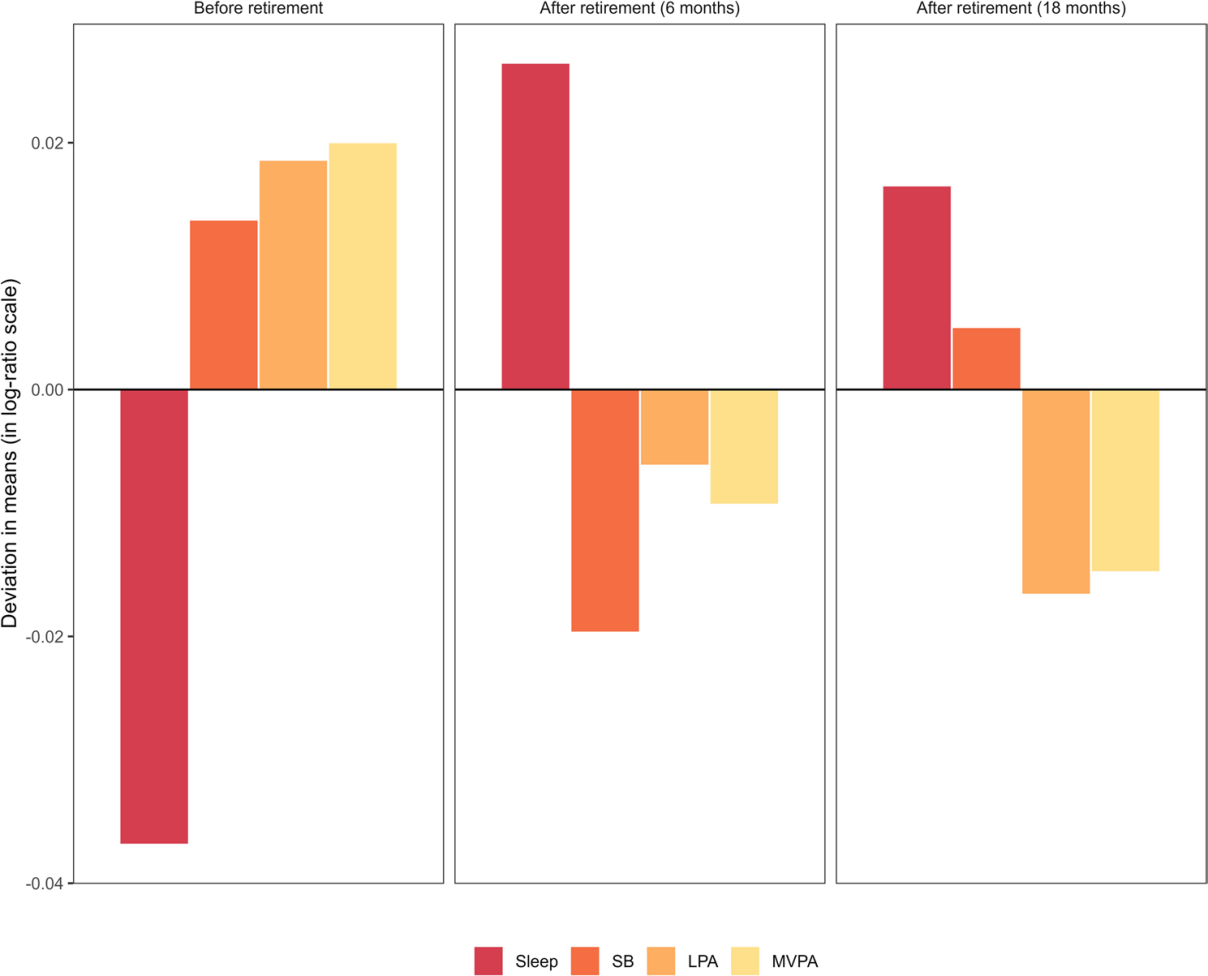
Geometric mean changes bar plots by retirement status

Figure 1 shows geometric mean changes across retirement. Bars, in log-ratio scale to facilitate interpretation, illustrate the extent and sign of the difference (in mean) of each movement behavior relative to the reference line at zero, which represents the overall mean. Sleep time, which was below average during pre-retirement, increased markedly post-retirement, especially in the 6 months post-retirement follow-up. In contrast, SB was relatively lower in the first post-retirement period. Compared to the pre-retirement movement behavior profile, LPA was relatively lower after retirement, particularly at the 18 months follow-up. Similarly, average post-retirement MVPA time decreased markedly.

### Retirement and movement behavior analysis

Following the CoDA methodology, the original 4-part composition was represented using 3 ilr-coordinates $\:{ilr}_{1}$, $\:{ilr}_{2}$ and $\:{ilr}_{3}$, as previously noted. Linear mixed models were then fitted to examine the effect of retirement status on movement behaviors. The results are summarized in Table 3. The first model (left column), where $\:{ilr}_{1}$ is the response variable, indicates that the balance of sleep over wake time increased significantly after retirement, in both the first (*β*: 0.08, 95% Confidence Interval (CI: 0.04–0.12) and the second follow-up (*β*: 0.07, 95% CI: 0.03–0.11). The second and third models (Table 3, middle and right columns) indicate that there were no discernible retirement effects in contrasts between SB and all physical activity and between LPA and MVPA.

**Table 3:** Estimates from mixed models of movement behavior ilr-coordinates on retirement status (*N* = 112)

	$\:{ilr}_{1}$- Sleep vs. Wake		$\:{ilr}_{2}$- SB vs. all PA		$\:{ilr}_{3}$- LPA vs. MVPA	
*Fixed factors*	*β (95% CI)*	*P-value*	*β (95% CI)*	*P-value*	*β (95% CI)*	*P-value*
Post-retirement (6 months)^a^	0.10 (0.05, 0.14)	**< 0.001**	0.03 (−0.05, 0.11)	0.458	−0.00 (−0.06, 0.06)	0.936
Post-retirement (18 months)^a^	0.08 (0.03, 0.14)	**0.004**	0.08 (−0.02, 0.18)	0.130	−0.01 (−0.09, 0.07)	0.782

Statistically significant P-values are shown in bold. All models are additionally adjusted for thefollowing covariates: gender, age, marital status, occupational social class, and life-affectingchronic diseases; the full model table with all covariates is shown in the appendix (Table A1). Reference categories: Before retirement (6 months). *Abbreviations*: *PA* Physical Activity, *CI* Confidence Interval

**Fig. 2:**
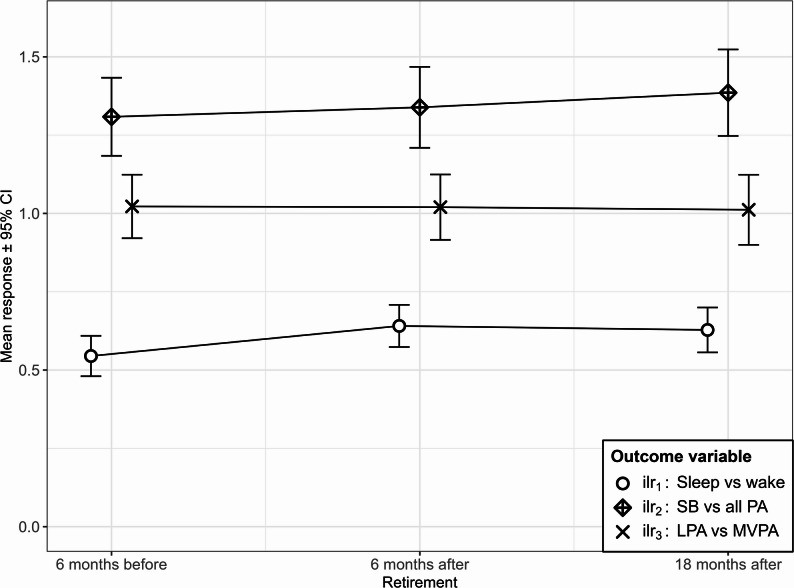
Marginal means from mixed models of movement behavior over retirement periods and OPA levels Notes: Error bars represent 95% confidence intervals. *PA* physical activity

Estimated marginal mean responses across retirement periods were computed from the models (Fig. 2). Pairwise post-hoc comparisons between them were conducted based on *t*-tests (Table A2). With respect to the balance between sleep and wake time ($\:{ilr}_{1}$) across the retirement transition, there was a significant difference between pre-retirement and 6 months and 18 months post-retirement (FDR-adjusted *p* < 0.001 and *p* = 0.005 respectively), which was not the case for the two post-retirement follow-ups (FDR-adjusted *p* = 0.596). Consistently, there were no meaningful differences between any pair of retirement periods for $\:{ilr}_{2}$ and $\:{ilr}_{3}$.

### Effect modification of pre-retirement OPA

**Fig. 3:**
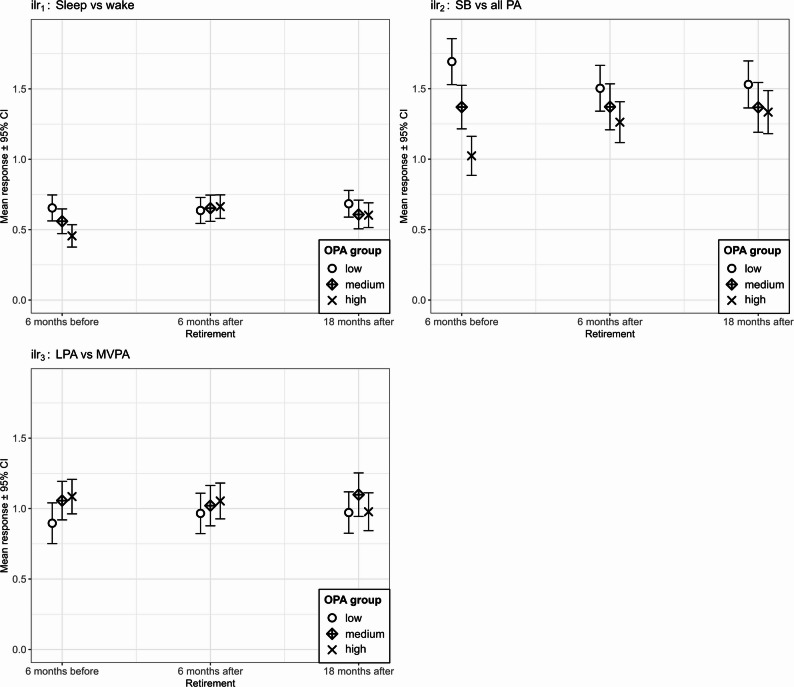
Marginal means from mixed models of movement behavior over retirement periods and OPA levelsError bars represent 95% confidence intervals. *PA* physical activity

Table 4 shows the results from the mixed models fitted to the movement behavior ilr-coordinates on retirement status, OPA tertile groups and the interaction between them. Main effects coefficients indicate that medium and high OPA groups, overall, had lower ratios of sleep over wake time – though to a lesser extent and not statistically significant for the medium OPA group – and of SB over all PA, compared to the low OPA group. On the contrary, the medium and high OPA groups had an overall higher LPA over MVPA ratio compared to the low OPA group. Significant interaction terms in the models suggest that changes in the three ilr variables across retirement differ according to OPA groups. These differences in movement behaviors across retirement by OPA group are further explored and tested with the marginal means plots (Fig. 3) and pairwise comparison tests (Table A4).

**Table 4:** Estimates from mixed models of movement behavior on retirement status and OPA (*N* = 112)

	$\:{ilr}_{1}$- Sleep vs. Wake		$\:{ilr}_{2}$- SB vs. all PA		$\:{ilr}_{3}$- LPA vs. MVPA	
*Fixed factors*	*β (95% CI)*	*P-value*	*β (95% CI)*	*P-value*	*β (95% CI)*	*P-value*
OPA medium^a^	−0.09 (−0.19, 0.00)	0.058	−0.32 (−0.49, −0.15)	**< 0.001**	0.16 (0.01, 0.31)	**0.034**
OPA high^a^	−0.20 (−0.30, −0.10)	**< 0.001**	−0.67 (−0.84, −0.49)	**< 0.001**	0.19 (0.03, 0.34)	**0.017**
Post-retirement (6 months)^b^	−0.02 (−0.09, 0.05)	0.610	−0.19 (−0.30, −0.08)	**< 0.001**	0.07 (−0.02, 0.16)	0.110
Post-retirement (18 months)^b^	0.03 (−0.05, 0.11)	0.457	−0.16 (−0.29, −0.04)	**0.013**	0.08 (−0.03, 0.18)	0.141
OPA medium × 6 mn post-retirement	0.11 (0.01, 0.21)	**0.026**	0.19 (0.04, 0.34)	**0.015**	−0.11 (−0.22, 0.01)	0.074
OPA high × 6 mn post-retirement	0.23 (0.13, 0.32)	**< 0.001**	0.43 (0.28, 0.58)	**< 0.001**	−0.10 (−0.21, 0.01)	0.080
OPA medium × 18 mn post-retirement	0.02 (−0.09, 0.12)	0.726	0.16 (0.00, 0.32)	0.054	−0.03 (−0.16, 0.09)	0.591
OPA high × 18 mn post-retirement	0.12 (0.02, 0.22)	**0.020**	0.48 (0.32, 0.63)	**< 0.001**	−0.18 (−0.30, −0.07)	**0.002**

Statistically significant P-values are shown in bold. All models are additionally adjusted for thefollowing covariates: gender, age, marital status, occupational social class, and life-affectingchronic diseases; the full model table with all covariates is shown in the appendix (Table A1). Reference categories: Before retirement (6 months). *Abbreviations*: *PA * Physical Activity, *mm months, CI * Confidence Interval

When considering $\:{ilr}_{1}$ differences across retirement within OPA groups (Fig. 3, top-right panel), the high OPA group significantly increases the sleep to wake time ratio, as supported by comparison tests between the pre-retirement period with the two first post-retirement follow-ups (FDR-adjusted *p* < 0.001 and *p* < 0.001). A significant, albeit much smaller, increase in the $\:{ilr}_{1}$ ratio was also observed for the medium OPA between the pre-retirement period and the 6-month post-retirement follow-up (FDR-adjusted *p* = 0.045).

Regarding the $\:{ilr}_{2}$ ratio, the low OPA group decreased SB to physical activity ratio in the post-retirement follow-ups relative to pre-retirement (FDR-adjusted *p* = 0.003 and *p* = 0.019), while the high OPA group increased it (FDR-adjusted *p* < 0.001 and *p* < 0.001). This led the two groups to broadly converge in the post-retirement periods (Fig. 3, top-right panel). For the medium OPA group, the balance between SB and all physical activity was relatively stable across retirement.

In the $\:{ilr}_{3}$ model, where a significant interaction was only found for the high OPA group and the 18-month post-retirement period (Table 4), differences between groups were less clear based on estimated marginal means (Fig. 3, bottom-left panel). Furthermore, none of the pairwise comparisons within OPA groups across retirement were statistically significant (Table A4). Finally, all three ilr balances remained approximately stable after retirement for all OPA groups, as no statistically significant differences were found in the pairwise comparisons between the two post-retirement periods (Table A4).

## Discussion

This study used a CoDA approach to examine the change in 24-hour movement behaviors across the retirement transition. This is the first study to examine the modifying role of pre-retirement OPA on changes in device-measured 24-hour movement behavior over the retirement transition. In the overall sample sleep time increased relative to wake time, whereas changes in SB relative to all physical activity, and in LPA relative to MVPA, were not detected. However, changes in SB and physical activity were observed when examining subgroups with different levels of pre-retirement OPA. There appeared to be a convergence between OPA groups across retirement, where those with high OPA increased the SB to all physical activity ratio, while those with low OPA decreased it. To a smaller extent, also the pre-retirement differences in sleep relative to wake time and in LPA relative to MVPA between the low and high OPA groups reduced post-retirement. While the high OPA group had low sleep relative to wake time and low LPA relative to MVPA, these ratios increased to comparable levels as the low OPA group.

Our findings corroborate previous research showing an increase in sleep duration across retirement [30, 44, 45]. Our findings add nuance to this literature by indicating that individuals with high pre-retirement OPA underlie the increase in sleep time at the population level. Proposed mechanisms for the beneficial effect of retirement on sleep, include the removal of stress and scheduling constraints associated with paid work [45]. In this regard, those with higher OPA may have jobs with work schedules that hinder sufficient sleep; these individuals may stand to benefit more from the rest and recovery afforded by retirement.

Turning to physical activity and SB, our results suggest that the extent of physical activity during work periods is an important factor in activity changes across retirement. This corroborates a previous study, which indicated that self-reported physical activity changes over retirement were dependent on the physical demands of the occupation [4]. At the same time, these findings partly diverge from a previous study, where relative differences between LPA and MVPA were found independently of occupational class [27]. This divergence may be explained by different variables used to measure pre-retirement occupation, as our variable measured OPA, which reflects physical activity at work rather than occupational social class; and differences in type of device (i.e., wrist actigraphy instead of thigh accelerometer) [46].

Findings show that people with high OPA reduce their overall physical activity time in favor of SB during retirement. While this may seem like an “unhealthy” shift, it could be considered beneficial when viewed in light of the “physical activity paradox” and the “Sweet-Spot hypothesis” [14, 20], as this group reduces their potentially detrimental OPA while increasing sleep time. These changes in time use may represent a shift toward the high OPA group's particular “sweet-spot” of movement behaviors. Similarly, the post-retirement reduction in SB among individuals with low OPA may also represent a shift toward their particular “sweet-spot”, albeit in the opposite direction, as retirement may allow work-related SB to be replaced by recreational physically active time [9]. Since paid work, physical activity, SB and sleep are all time-dependent activities [47], retirement may allow individuals to adopt a healthier balance of movement behaviors once the barrier of work demands is removed.

The current study has several strengths and limitations. Due to the collection of different types of data and the CoDA approach, this study contributes a holistic view of changes in movement behaviors across retirement. These findings also add nuance to the role of pre-retirement OPA among older adults’ movement behaviors, which can be attributed to the useful integration of device-based measurements together with diary data on work spells. Additionally, the thigh accelerometer posture-based classification used here is considered to be more reliable to assess sedentary and physically active states compared to, for instance, wrist actigraphy [46]. The longitudinal design further allowed us to assess changes across the retirement transition in a sample of older adults, who have been underrepresented in studies of movement behaviors with device-based measurements.

In terms of limitations, the sample was relatively small. Future studies using larger samples to better assess population heterogeneity would be beneficial. In particular, socioeconomic status has been shown to be related to levels of occupational and leisure-time physical activity, suggesting that mechanisms related to socioeconomic inequalities may play a role in these associations [48]. Although we adjusted for occupational social class, a detailed exploration of socioeconomic differentials was outside the scope of this study due to the lack of a diverse representation of social strata in our sample. Similarly, variations in retirement pathways should also be examined in more detail, as individuals’ retirement plans may be related to socioeconomic and occupational characteristics [49]. While the SRS adopted a flexible definition of retirement, which reflects the current social reality where retirees often keep working, albeit for a considerably reduced time, future research could assess differences according to specific modes of retirement. Given the SRS selection criteria, which required participants to report intention to retire at a typical retirement age, it is likely that retirees followed more normative retirement trajectories and were selected in other respects, such as health and workability [31].

Another limitation is that, while the CoDA approach provides a holistic view of movement behaviors, limiting analyses to only four movement categories may hinder more fine-grained and meaningful classifications of time use. For example, we could not assess daytime naps, as sleep was restricted to nighttime periods. Additionally, information on commuting to and from work was not collected, which may play a role in measurements of occupational-related physical activity. Our findings are also not generalizable outside of Sweden.

## Conclusion

This study provides novel insights into changes in movement behaviors across retirement, highlighting the pivotal role of pre-retirement OPA in shaping these shifts. Using a compositional data analysis approach, we found that sleep duration increased significantly post-retirement, particularly among those with high OPA, while changes in sedentary behavior and physical activity were more nuanced and OPA-dependent. Notably, the convergence of movement patterns between high and low OPA groups after retirement suggests that retirement may serve as an equalizer, mitigating pre-existing disparities in movement behaviors. Our findings underscore the complexity of interpreting post-retirement activity changes: while reduced physical activity in high-OPA individuals might seem unfavorable, it could reflect a shift toward a healthier balance, which would align with recommendations based on the “Sweet-Spot hypothesis”. By contextualizing retirement as a dynamic transition with measurable effects on daily activity, this work contributes to a growing body of evidence supporting tailored health interventions for retirees, particularly those from physically demanding occupations.

## Supplementary Information

Supplementary Material 1.

## Data Availability

Given restrictions from the ethical review board and considering that sensitive personal data are involved, it is not possible to make the data freely available. Access to the data may be provided to other researchers in line with Swedish law and after consultation with the Stockholm University legal department. Requests for data, stored at the Stress Research Institute, Department of Psychology, should be sent to registrator@su.se with reference to the the Swedish Retirement Study or directly to the corresponding author.

## Abbreviations

SB: Sedentary Behavior
LPA: Light Physical Activity
MVPA: Moderate-Vigorous Physical Activity
OPA: Occupational Physical Activity
CoDA: Compositional Data Analysis
SRS: Swedish Retirement Study
SLOSH: Swedish Longitudinal Occupational Survey of Health
FDR: False Discovery Rate
ilr: isometric log-ratio
CI: Confidence Interval
MICE: Multiple imputation by chained equations
WHO: World Health Organization
OECD: Organisation for Economic Co-operation and Development
