# Supplementary material for: Changes in physical activity across retirement: a compositional data analysis approach in a Swedish cohort study
Source: Eur Rev Aging Phys Act. 2025 Dec 19;23:3. doi: 10.1186/s11556-025-00395-6 (PMC12829166; doi:10.1186/s11556-025-00395-6)
Supplement: Supplementary file 1 — Supplementary Material 1. [file 11556_2025_395_MOESM1_ESM.docx]

# Appendix

*Table A1 - Estimates from mixed models of movement behavior on retirement status (N=112).*

|  | ${ilr}_{1}$ - Sleep vs Wake | | | ${ilr}_{2}$ - SB vs all PA | | | ${ilr}_{3}$ - LPA vs MVPA | | |
| --- | --- | --- | --- | --- | --- | --- | --- | --- | --- |
| Covariates | β | 95% CI | P-value | β | 95% CI | P-value | β | 95% CI | P-value |
| Retirement |  |  |  |  |  |  |  |  |  |
| Pre-retirement | Ref. |  |  |  |  |  |  |  |  |
| Post-retirement (6 months) | 0.10 | 0.05, 0.14 | **<0.001** | 0.03 | -0.05, 0.11 | 0.458 | 0.00 | -0.06, 0.06 | 0.936 |
| Post-retirement (18 months) | 0.08 | 0.03, 0.14 | **0.004** | 0.08 | -0.02, 0.18 | 0.130 | -0.01 | -0.09, 0.07 | 0.782 |
| Gender |  |  |  |  |  |  |  |  |  |
| Men | Ref. |  |  |  |  |  |  |  |  |
| Women | -0.06 | -0.13, 0.01 | 0.103 | -0.18 | -0.32, -0.04 | **0.014** | 0.12 | 0.01, 0.24 | **0.039** |
| Age | -0.01 | -0.03, 0.01 | 0.424 | -0.02 | -0.06, 0.01 | 0.212 | 0.00 | -0.02, 0.03 | 0.746 |
| Marital status |  |  |  |  |  |  |  |  |  |
| Married/cohabiting | Ref. |  |  |  |  |  |  |  |  |
| Single or non-cohabiting | -0.09 | -0.19, 0.01 | 0.092 | 0.14 | -0.06, 0.35 | 0.168 | -0.05 | -0.21, 0.12 | 0.595 |
| Occupational social class |  |  |  |  |  |  |  |  |  |
| Non-manual, ISCO 1-4 | Ref. |  |  |  |  |  |  |  |  |
| Manual, ISCO 5-9 | 0.02 | -0.07, 0.11 | 0.671 | 0.05 | -0.14, 0.23 | 0.622 | 0.27 | 0.12, 0.42 | **<0.001** |
| Chronic disease(s) affecting life |  |  |  |  |  |  |  |  |  |
| none | Ref. |  |  |  |  |  |  |  |  |
| 1 chronic disease | 0.05 | 0.00, 0.10 | 0.070 | 0.05 | -0.05, 0.14 | 0.321 | 0.05 | -0.02, 0.11 | 0.158 |
| >1 chronic diseases | 0.05 | -0.01, 0.11 | 0.111 | 0.08 | -0.03, 0.18 | 0.143 | 0.02 | -0.06, 0.09 | 0.675 |
| Notes: ILR = Isometric Log-Ratios; PA = Physical Activity, CI = Confidence Interval, Ref.=Reference category.  Statistically significant P-values in bold. | | | | | | | | | |

Table A2 –Pairwise contrasts from mixed models estimates.

| Pairwise contrasts | ilr1 - Sleep vs Wake | | ilr2 - SB vs all PA | | ilr3 - LPA vs MVPA | |
| --- | --- | --- | --- | --- | --- | --- |
|  | Est. Diff. | p-value | Est. Diff. | p-value | Est. Diff. | p-value |
| 6mn pre - 6mn post | -0.096 | **<0.001** | -0.030 | 0.459 | 0.002 | 0.936 |
| 6mn pre - 18mn post | -0.083 | **0.005** | -0.077 | 0.385 | 0.011 | 0.936 |
| 6mn post - 18mn post | 0.013 | 0.596 | -0.047 | 0.385 | 0.008 | 0.936 |

Notes: Est. Diff. = Estimated Differences in marginal means, PA = physical activity, mn = Months,
pre = Pre-retirement, post = Post-retirement. Estimates represent pairwise comparisons with Benjamini-Hockberg correction for false discovery rate (FDR). Statistically significant P-values in bold.

Table A3 - Estimates from mixed models of movement behavior on retirement status and OPA (N=112).

|  | ${ilr}_{1}$ - Sleep vs Wake | | | ${ilr}_{2}$ - SB vs all PA | | | ${ilr}_{3}$ - LPA vs MVPA | | |
| --- | --- | --- | --- | --- | --- | --- | --- | --- | --- |
| Covariates | β | 95% CI | P-value | β | 95% CI | P-value | β | 95% CI | P-value |
| OPA tertiles |  |  |  |  |  |  |  |  |  |
| Low | Ref. |  |  |  |  |  |  |  |  |
| Medium | -0.09 | -0.19, 0.00 | 0.058 | -0.32 | -0.49, -0.15 | **<0.001** | 0.16 | 0.01, 0.31 | **0.034** |
| High | -0.20 | -0.30, -0.10 | **<0.001** | -0.67 | -0.84, -0.49 | **<0.001** | 0.19 | 0.03, 0.34 | **0.017** |
| Retirement |  |  |  |  |  |  |  |  |  |
| Pre-retirement | Ref. |  |  |  |  |  |  |  |  |
| Post-retirement (6 months) | -0.02 | -0.09, 0.05 | 0.610 | -0.19 | -0.30, -0.08 | **<0.001** | 0.07 | -0.02, 0.16 | 0.110 |
| Post-retirement (18 months) | 0.03 | -0.05, 0.11 | 0.457 | -0.16 | -0.29, -0.04 | **0.013** | 0.08 | -0.03, 0.18 | 0.141 |
| Gender |  |  |  |  |  |  |  |  |  |
| Men | Ref. |  |  |  |  |  |  |  |  |
| Women | -0.04 | -0.11, 0.04 | 0.322 | -0.08 | -0.22, 0.05 | 0.232 | 0.09 | -0.03, 0.21 | 0.143 |
| Age | 0.00 | -0.02, 0.01 | 0.662 | -0.01 | -0.04, 0.02 | 0.531 | 0.00 | -0.03, 0.03 | 0.996 |
| Marital status |  |  |  |  |  |  |  |  |  |
| Married/cohabiting | Ref. |  |  |  |  |  |  |  |  |
| Single or non-cohabiting | -0.09 | -0.19, 0.02 | 0.096 | 0.15 | -0.03, 0.34 | 0.105 | -0.04 | -0.21, 0.13 | 0.620 |
| Occupational social class |  |  |  |  |  |  |  |  |  |
| Non-manual, ISCO 1-4 | Ref. |  |  |  |  |  |  |  |  |
| Manual, ISCO 5-9 | 0.05 | -0.05, 0.15 | 0.301 | 0.19 | 0.01, 0.38 | 0.037 | 0.24 | 0.07, 0.40 | **0.004** |
| Chronic disease(s) affecting life |  |  |  |  |  |  |  |  |  |
| none | Ref. |  |  |  |  |  |  |  |  |
| 1 chronic disease | 0.04 | -0.01, 0.09 | 0.110 | 0.04 | -0.05, 0.12 | 0.406 | 0.05 | -0.01, 0.12 | 0.105 |
| >1 chronic diseases | 0.05 | -0.01, 0.11 | 0.103 | 0.07 | -0.03, 0.16 | 0.164 | 0.02 | -0.06, 0.09 | 0.685 |
| OPA tertiles × Retirement |  |  |  |  |  |  |  |  |  |
| OPA medium ×   6 months post-retirement | 0.11 | 0.01, 0.21 | **0.026** | 0.19 | 0.04, 0.34 | **0.015** | -0.11 | -0.22, 0.01 | 0.074 |
| OPA high ×   6 months post-retirement | 0.23 | 0.13, 0.32 | **<0.001** | 0.43 | 0.28, 0.58 | **<0.001** | -0.10 | -0.21, 0.01 | 0.080 |
| OPA medium ×   18 months post-retirement | 0.02 | -0.09, 0.12 | 0.726 | 0.16 | 0.00, 0.32 | 0.054 | -0.03 | -0.16, 0.09 | 0.591 |
| OPA high ×   18 months post-retirement | 0.12 | 0.02, 0.22 | **0.020** | 0.47 | 0.32, 0.63 | **<0.001** | -0.18 | -0.30, -0.07 | **0.002** |
| Notes: PA = Physical Activity, CI = Confidence Interval, Ref.=reference category. Statistically significant P-values in bold. | | | | | | | | | |

Table A4 – Pairwise contrasts from mixed models estimates.

| Pairwise contrasts | ilr1 - Sleep vs Wake | | ilr2 - SB vs all PA | | ilr3 - LPA vs MVPA | |
| --- | --- | --- | --- | --- | --- | --- |
|  | Est. Diff. | P-value | Est. Diff. | P-value | Est. Diff. | P-value |
| Low OPA | | | | | | |
| 6mn pre - 6mn post | 0.0182 | 0.610 | 0.1893 | **0.003** | -0.0698 | 0.211 |
| 6mn pre - 18mn post | -0.0297 | 0.610 | 0.1619 | **0.019** | -0.0758 | 0.211 |
| 6mn post - 18mn post | -0.0479 | 0.581 | -0.0274 | 0.637 | -0.0059 | 0.894 |
| Medium OPA | | | | | | |
| 6mn pre - 6mn post | -0.0926 | **0.045** | -0.0018 | 0.979 | 0.0356 | 0.442 |
| 6mn pre - 18mn post | -0.0482 | 0.281 | 0.0018 | 0.979 | -0.0421 | 0.442 |
| 6mn post - 18mn post | 0.0444 | 0.281 | 0.0037 | 0.979 | -0.0778 | 0.355 |
| High OPA | | | | | | |
| 6mn pre - 6mn post | -0.2079 | **<0.001** | -0.2392 | **<0.001** | 0.0310 | 0.484 |
| 6mn pre - 18mn post | -0.1471 | **<0.001** | -0.3104 | **<0.001** | 0.1075 | 0.110 |
| 6mn post - 18mn post | 0.0608 | 0.103 | -0.0713 | 0.227 | 0.0766 | 0.136 |

Notes: Est. Diff. = Estimated Differences in marginal means, PA = physical activity, mn = Months,
pre = Pre-retirement, post = Post-retirement. Estimates represent pairwise comparisons with Benjamini-Hockberg correction for false discovery rate (FDR). Statistically significant P-values in bold.
